# Genome-wide association meta-analysis of circulating odd-numbered chain saturated fatty acids: Results from the CHARGE Consortium

**DOI:** 10.1371/journal.pone.0196951

**Published:** 2018-05-08

**Authors:** Marcia C. de Oliveira Otto, Rozenn N. Lemaitre, Qi Sun, Irena B. King, Jason H. Y. Wu, Ani Manichaikul, Stephen S. Rich, Michael Y. Tsai, Y. D. Chen, Myriam Fornage, Guan Weihua, Stella Aslibekyan, Marguerite R. Irvin, Edmond K. Kabagambe, Donna K. Arnett, Majken K. Jensen, Barbara McKnight, Bruce M. Psaty, Lyn M. Steffen, Caren E. Smith, Ulf Risérus, Lars Lind, Frank B. Hu, Eric B. Rimm, David S. Siscovick, Dariush Mozaffarian

**Affiliations:** 1 Division of Epidemiology, Human Genetics and Environmental Sciences, the University of Texas Health Science Center, School of Public Health, Houston, TX, United States of America; 2 Cardiovascular Health Research Unit, Department of Medicine, University of Washington, Seattle, WA, United States of America; 3 Department of Nutrition and Epidemiology, Harvard T.H. Chan School of Public Health and Channing Division of Network Medicine, and Harvard Medical School, Boston, MA, United States of America; 4 Brigham and Women’s Hospital and Harvard Medical School, Boston, MA, United States of America; 5 University of New Mexico, Albuquerque, NM, United States of America; 6 The George Institute for Global Health and the Faculty of Medicine, University of New South Wales, Sydney, Australia; 7 Center for Public Health Genomics, University of Virginia, Charlottesville, VA, United States of America; 8 Department of Laboratory Medicine and Pathology, University of Minnesota, Minneapolis, MN, United States of America; 9 Institute for Translational Genomics and Population Sciences, Los Angeles Biomedical Research Institute at Harbor, UCLA Medical Center, Torrance, CA, United States of America; 10 Key Laboratory of Nutrition and Metabolism, the University of Texas Health Science Center, School of Public Health, Houston, TX, United States of America; 11 Department of Biostatistics, University of Minnesota, Minneapolis, MN, United States of America; 12 College of Public Health, University of Kentucky, Lexington, KY, United States of America; 13 Department of Nutrition and Epidemiology, Harvard T.H. Chan School of Public Health, Boston MA, United States of America; 14 Department of Biostatistics, University of Washington, Seattle, WA, United States of America; 15 Kaiser Permanente Washington Health Research Institute, Seattle, WA, United States of America; 16 School of Public Health, Division of Epidemiology and Community Health, University of Minnesota, Minneapolis, Minnesota, United States of America; 17 Nutrition and Genomics Laboratory, Jean Mayer USDA HNRCA at Tufts University, Boston, MA, United States of America; 18 Department of Public Health and Caring Sciences, Uppsala University, Uppsala, Sweden; 19 Department of Medical Sciences, Uppsala University, Uppsala, Sweden; 20 The New York Academy of Medicine, New York, NY, United States of America; 21 Friedman School of Nutrition Science and Policy, Tufts University, Boston, MA, United States of America; University of Illinois, UNITED STATES

## Abstract

**Background:**

Odd-numbered chain saturated fatty acids (OCSFA) have been associated with potential health benefits. Although some OCSFA (e.g., C15:0 and C17:0) are found in meats and dairy products, sources and metabolism of C19:0 and C23:0 are relatively unknown, and the influence of non-dietary determinants, including genetic factors, on circulating levels of OCSFA is not established.

**Objective:**

To elucidate the biological processes that influence circulating levels of OCSFA by investigating associations between genetic variation and OCSFA.

**Design:**

We performed a meta-analysis of genome-wide association studies (GWAS) of plasma phospholipid/erythrocyte levels of C15:0, C17:0, C19:0, and C23:0 among 11,494 individuals of European descent. We also investigated relationships between specific single nucleotide polymorphisms (SNPs) in the lactase (*LCT*) gene, associated with adult-onset lactase intolerance, with circulating levels of dairy-derived OCSFA, and evaluated associations of candidate sphingolipid genes with C23:0 levels.

**Results:**

We found no genome-wide significant evidence that common genetic variation is associated with circulating levels of C15:0 or C23:0. In two cohorts with available data, we identified one intronic SNP (rs13361131) in myosin X gene (*MYO10)* associated with C17:0 level (P = 1.37×10^−8^), and two intronic SNP (rs12874278 and rs17363566) in deleted in lymphocytic leukemia 1 (*DLEU1)* region associated with C19:0 level (P = 7.07×10^−9^). In contrast, when using a candidate-gene approach, we found evidence that three SNPs in *LCT* (rs11884924, rs16832067, and rs3816088) are associated with circulating C17:0 level (adjusted P = 4×10^−2^). In addition, nine SNPs in the ceramide synthase 4 (*CERS4)* region were associated with circulating C23:0 levels (adjusted P<5×10^−2^).

**Conclusions:**

Our findings suggest that circulating levels of OCSFA may be predominantly influenced by non-genetic factors. SNPs associated with C17:0 level in the *LCT* gene may reflect genetic influence in dairy consumption or in metabolism of dairy foods. SNPs associated with C23:0 may reflect a role of genetic factors in the synthesis of sphingomyelin.

## Introduction

The odd-numbered chain saturated fatty acids (OCSFA), i.e., pentadecanoic acid (C15:0) and heptadecanoic acid (C17:0), are found in ruminant foods such as meats or dairy products synthesized by the bacterial flora in the rumen [1] and seafood [2]. Multiple observational studies have suggested potential health benefits of higher circulating C15:0 and C17:0, such as lower risk of type 2 diabetes and cardiovascular disease [3–6], and improvement of risk factors such as blood pressure, plasma triglycerides, and insulin resistance [3, 7]. Based on the hypothesis that OCSFA cannot be synthesized by humans, circulating levels of C15:0 and C17:0 have been used as objective markers of dairy fat consumption [7–13]. However, the correlation between self-reported dairy fat consumption and levels of C15:0 or C17:0 has been modest [3, 14, 15], raising questions as to whether intrinsic genetic factors may influence OCSFA incorporation, metabolism or competition with other fatty acids (FA); whether self-reported intakes do not accurately capture true consumption of dairy fat, for instance due to many hidden sources (e.g., from milk, cream, butter) in numerous mixed dishes, bakery products, and processed and packaged foods [6], or alternatively whether other dietary sources, such as seafood, also contribute to circulating levels of these FA [2]. Genetic factors could also influence dietary consumption; for example, single nucleotide polymorphisms (SNPs) associated with reduced lactose tolerance could influence dairy intake and thereby circulating levels of OCSFA. Yet, the effects of common genetic variation on levels of C15:0 and C17:0 are not well-established.

In addition to C15:0 and C17:0, trace OCSFA, such as nonadecanoic acid (C19:0) and tricosanoic acid (C23:0), are found in the circulation, the sources and metabolism of which are relatively unknown. No prior studies, to our knowledge, have assessed genetic determinants of circulating levels of C19:0 or C23:0.

To elucidate the genetic factors influencing circulating OCSFA, we performed a genome-wide association studies (GWAS) meta-analysis of plasma phospholipid/erythrocyte C15:0, C17:0, C19:0, and C23:0 levels obtained from up to 11,494 individuals of European descent, as part of the Cohorts for Heart and Aging Research in Genomic Epidemiology (CHARGE) Consortium. We also investigated the association between SNPs in lactase (*LCT*), a gene associated with lactose intolerance [16, 17], with circulating levels of dairy-derived OCSFA. Finally, we examined the association of C23:0 levels with SNPs in sphingolipid genes previously associated with other very long chain saturated fatty acids (VLSFA) [18].

## Materials and methods

### Populations

We conducted a collaborative consortium investigation using data from 8 cohorts participating in the CHARGE Fatty Acid Working Group, comprising 11,494 individuals of European descent (**Table 1**). Participating cohorts included the Atherosclerosis Risk in Communities (ARIC) Study, the Coronary Artery Risk Development in Young Adults (CARDIA) Study, the Cardiovascular Health Study (CHS), the Genetics of Lipid-Lowering Drugs and Diet Network (GOLDN), the Health Professionals Follow-up Study (HPFS), the Multi-Ethnic Study of Atherosclerosis (MESA), the Nurses’ Health Study (NHS), and the Prospective Investigation of the Vasculature in Uppsala Seniors (PIVUS). Details of participating cohorts are presented in the S1 Text. All participants provided informed written consent, including consent to participate in genetic studies; and all studies received approval from local ethical oversight committees. This meta-analysis does not qualify as human subject research since no access to identifiable private information was granted from any participating cohort.

**Table 1:** Characteristics of study participants in the participating cohorts.

Cohort^1^	N	Age (years)	% Women	FA Level (% of total FA)^2^				
				C15:0	C17:0	C15:0+C17:0	C19:0	C23:0
ARIC	3,269	53.8 (5.6)	51.3	0.17 (0.04)	NA	NA	NA	0.25 (0.07)
CARDIA	1,507	45.6 (3.3)	53.3	0.20 (0.06)	NA	NA	NA	NA
CHS	2,403	75.0 (5.1)	61.5	0.16 (0.04)	0.40 (0.07)	0.56 (0.10)	NA	0.75 (0.13)
GOLDN	774	48.6 (16.1)	50.4	1.08 (0.53)	0.36 (0.05)	1.44 (0.53)	NA	NA
HPFS	1,255	64.1 (8.5)	0	0.11 (0.05)	0.37 (0.10)	0.48 (0.14)	0.12 (0.05)	0.28 (0.05)
MESA	702	61.6 (10.4)	53.3	0.19 (0.05)	NA	NA	NA	NA
NHS	655	59.8 (6.5)	100	0.12 (0.04)	0.37 (0.08)	0.49 (0.11)	0.15 (0.09)	0.26 (0.06)
PIVUS	929	70.2 (0.16)	50.3	0.27 (0.08)	0.41 (0.07)	0.68 (0.13)	NA	NA

^1^Cohorts for Heart and Aging Research in Genomic Epidemiology (CHARGE) Consortium; Atherosclerosis Risk in Communities (ARIC) Study, the Coronary Artery Risk Development in Young Adults (CARDIA) Study, the Cardiovascular Health Study (CHS), the Genetics of Lipid-Lowering Drugs and Diet Network (GOLDN), the Health Professionals Follow-up Study (HPFS), the Multi-Ethnic Study of Atherosclerosis (MESA), the Nurses’ Health Study (NHS), and the Prospective Investigation of the Vasculature in Uppsala Seniors (PIVUS)

^2^Values in the table are mean (standard deviation) except where specified otherwise. NA: not available. FA were measured in erythrocyte membrane phospholipids (GOLDN, HPFS, NHS) and plasma phospholipids (ARIC, CARDIA, CHS, MESA and PIVUS).

### Measurements of phospholipid FA

FA were measured as % of total FA in plasma phospholipids in ARIC, CARDIA, CHS, MESA and PIVUS, and in erythrocyte membrane phospholipids in HPFS, GOLDN, and NHS. FA levels in plasma phospholipids and erythrocyte phospholipids are correlated, with reported correlations of 0.54 for C15:0 and 0.66 for C17:0 between these compartments [19]. Details of the FA measurement methods in each study are provided in the S1 Text. We evaluated each OCSFA separately, and also the combined levels of C15:0+C17:0.

### Genotyping and genome wide association analysis

Genotyping was performed separately in each cohort using high-density SNP marker platforms (ARIC, CARDIA, GOLDN and MESA: Affymetrix 6.0; CHS: Illumina 370; HPFS and NHS: Illumina 550k, 610Q, 660Q, Affymetrix 6.0; PIVUS: Illumina OmniExpress and CardioMetabochip). Samples with call rates <95–97% at genotyped markers were excluded. Genotypes were imputed to approximately 2.5 million HapMap SNPs by using either BEAGLE[20] (CARDIA), BIMBAM[21] (CHS), IMPUTE[22] (MESA, PIVUS), or MACH[23] (ARIC, GOLDN, HPFS, NHS). Compared to 1000G imputation, HapMap imputation allows similar identification of common variants when using appropriate Bonferroni correction [24]. SNPs for which Hardy-Weinberg equilibrium testing resulted in significant deviations from expectation (P*<*10^−4^ to <10^−6^, cohort-specific) were excluded from imputation. Additional details on genotyping and imputation in each cohort are provided in S1 Text.

We reviewed the influence of the number of measured FA in the assay on the relative concentrations of different FA. While the influence appeared modest to small, we evaluated association between SNP genotype and each FA separately within each cohort, quantifying change in FA levels associated with for each copy of specific alleles within each assay, in order to minimize any potential influence of other FA in the quantification of circulating OCSFA concentrations. All studies conducted linear regression analysis measuring the additive effect of the number of effect alleles, or equivalently the imputed number of effect alleles for imputed genotypes. In absence of a known model, we chose the additive model *a priori* as it has good power for all "monotone" models, including recessive and dominant [25]. The analyses used robust standard errors and adjusted for age, sex, site of recruitment where appropriate, and where needed, principal components to account for possible population genetic substructure.

### Meta-analysis

For each SNP and FA, study-specific GWAS results were combined using inverse-variance weighted meta-analysis using METAL (http://www.sph.umich.edu/csg/abecasis/metal). SNPs with minor allele frequency (MAF) ≤1% or imputation quality <0.30 were excluded from the meta-analyses. Genomic control correction was applied to each study prior to the meta-analysis and correction factors ranged from 0.98 to 1.08 (C15:0), 0.98 to 1.12 (C17:0), 0.98 to 1.07 (C15:0+C17:0), 0.97 to 0.98 (C19:0) and 1.00 to1.02 (C23:0). Associations between SNP and OCSFA were considered as “genome-wide significant” with P < 5×10^−8^.

We examined association of C15:0, C17:0 and C15:0+C17:0 levels *in silico* with SNPs in the *LCT* gene. We also evaluated the association between level of C23:0 with SNPs in ceramide synthase 4 (*CERS4)*, and serine palmitoyltransferase long chain base subunit 3 gene (*SPTLC3)*, two genes in the sphingolipid *de novo* biosynthesis pathway that had reported association with VLSFA levels [18]. Control of the false discovery rate (FDR) at 0.05 was applied to association between FA and SNPs in the candidate genes using the Benjamini and Hochberg method [26](SAS PROC MULTTEST procedure, SAS version 9.4). FDR-adjusted P < 5×10^−2^ were considered as statistically significant [27].

## Results

The eight participating cohorts included 11,494 subjects of European ancestry (Table 1), with mean age range at the time of FA measurement between 45 and 75 years (Table 1). Data availability varied by FA, ranging from two cohorts with data available on circulating C19:0 level to eight cohorts providing data on circulating C15:0 level.

### Genome wide associations with circulating odd chain saturated FA

In GWAS meta-analyses, no SNP associations with C15:0 level attained genome-wide significance (Table A in S1 Tables). The most strongly associated SNPs were located on chromosome 18, rs973730 (a synonymous SNP in establishment of sister chromatid cohesion N-acetyltransferase 1 gene (*ESCO1)*, P = 6.50×10^−7^), rs10502435 (2.2kb 3’ of *ESCO1*, P = 6.55×10^−7^), and rs12373434 (an intronic SNP in the establishment of sister chromatid cohesion N-acetyltransferase 1 gene (*ESCO1)*, P = 6.84×10^−7^). The three SNPs rs973730, rs10502435 and rs12373434 are in high linkage disequilibrium (r^2^ = 1).

For C17:0 level, an infrequent (MAF = 1.1%) SNP in the myosin X gene (*MYO10)*, rs13361131, attained genome-wide significance (P = 1.4×10^−8^) in analyses limited to the two cohorts with data on this SNP. Each copy of the rs13361131 G allele was associated with 0.14 percent of total FA higher level of C17:0 (Table 2). Compared to the mean level of C17:0 of 0.39 percent (weighted based on sample size, Table 2), this represented 36% higher level of C17:0 for each copy of the G allele. The rs7719940 SNP in *MYO10* is common (MAF = 27.9%) and its association with circulating levels of C15:0+C17:0 approached genome wide significance, (P = 7.1×10^−8^), although not its association with C15:0 level (P> 1.0×10^−4^) or C17:0 level (P = 9.65 × 10^−7^) individually (Tables A-C in S1 Tables).

**Table 2:** SNPs significantly associated with circulating OCSFA levels in European-ancestry participants.

Fatty Acid	Chromosome	Gene	SNP	Coded allele (frequency)	P value	Beta coefficient (SE)
**Genome wide associations**^**1**^						
**C17:0**	5	*MYO10*	rs13361131	A/G (0.99)	1.37×10^−8^	0.1346 (0.0237)
**C19:0**	13	*DLEU1*	rs12874278	T/C (0.06)	7.07×10^−9^	0.0199 (0.0034)
	13	*DLEU1*	rs17363566	A/G (0.06)	7.07×10^−9^	0.0199 (0.0034)
**Candidate gene associations**^**2**^						
**C17:0**	2	*LCT*	rs11884924	A/C (0.02)	4.3×10^−2^	-0.0208 (0.0068)
			rs16832067	G/A (0.98)	4.3×10^−2^	-0.0204 (0.0068)
			rs3816088	C/G (0.02)	4.3×10^−2^	-0.0201 (0.0068)
**C23:0**	19	*CERS4*	rs36251	C/G (0.55)	3.0 ×10^−2^	0.005 (0.002)
			rs10409603	A/G (0.44)	3.0 ×10^−2^	-0.0041 (0.001)
			rs2927718	C/G (0.42)	3.0 ×10^−2^	0.0036 (0.001)
			rs1115199	T/C (0.48)	3.0 ×10^−2^	-0.0041 (0.001)
			rs367443	A/G (0.46)	3.0 ×10^−2^	-0.004 (0.001)
			rs2306199	T/G (0.58)	3.0 ×10^−2^	-0.0042 (0.002)
			rs36249	A/G (0.44)	3.0 ×10^−2^	0.004 (0.001)
			rs36258	A/G (0.53)	3.0 ×10^−2^	0.0045 (0.002)
			rs17160348	T/C (0.16)	3.0 ×10^−2^	-0.0069 (0.003)

^1^Evaluated at genome-wide significance (alpha = 5.0×10^−8^).

^2^Evaluated at adjusted false discovery rate (alpha = 0.05).

**Fig 1:**
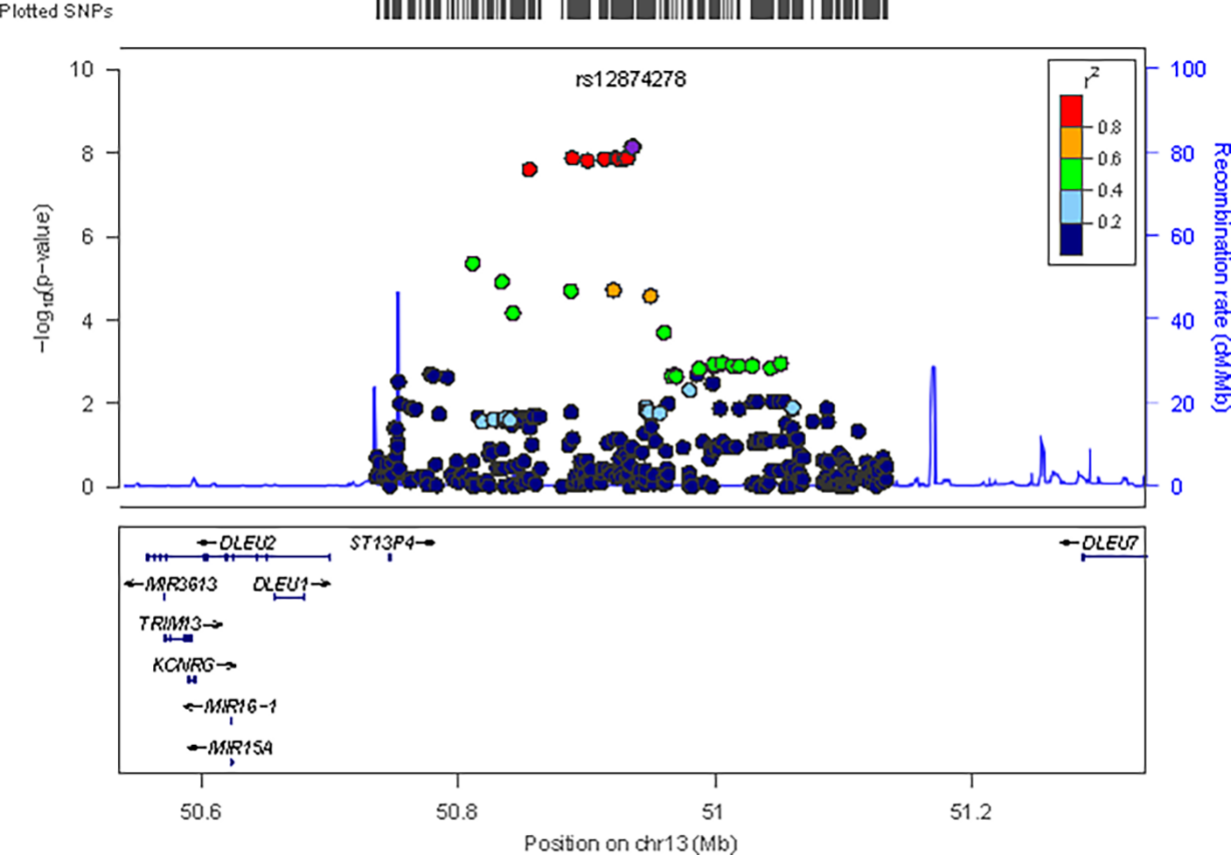
Single-nucleotide polymorphism (SNP) association plots for C19:0-associated region. Genetic coordinates are along the x axis, and genome-wide association significance level is plotted against the y axis as–log10 (P value). Linkage disequilibrium (LD) is indicated by color scale in relationship to marker rs12874278.

For C19:0 level, two common (MAF = 5.9%) intronic SNP in the deleted in lymphocytic leukemia 1 gene *(DLEU1)*, rs12874278 and rs17363566, achieved genome-wide significance (P = 7.1 × 10^−9^). Each copy of the T allele was associated with a 0.02 percent of total FA higher level of C19:0 (Table 2, Fig 1, and Table D in S1 Tables); or about a 15.4% higher level compared to the weighted mean level of 0.13 in the two cohorts with C19:0. For C23:0 level, no genome-wide significant association with any SNP was identified (Table E in S1 Tables).

### Associations between common SNPs in the *LCT* gene and biomarkers of dairy intake

**Fig 2:**
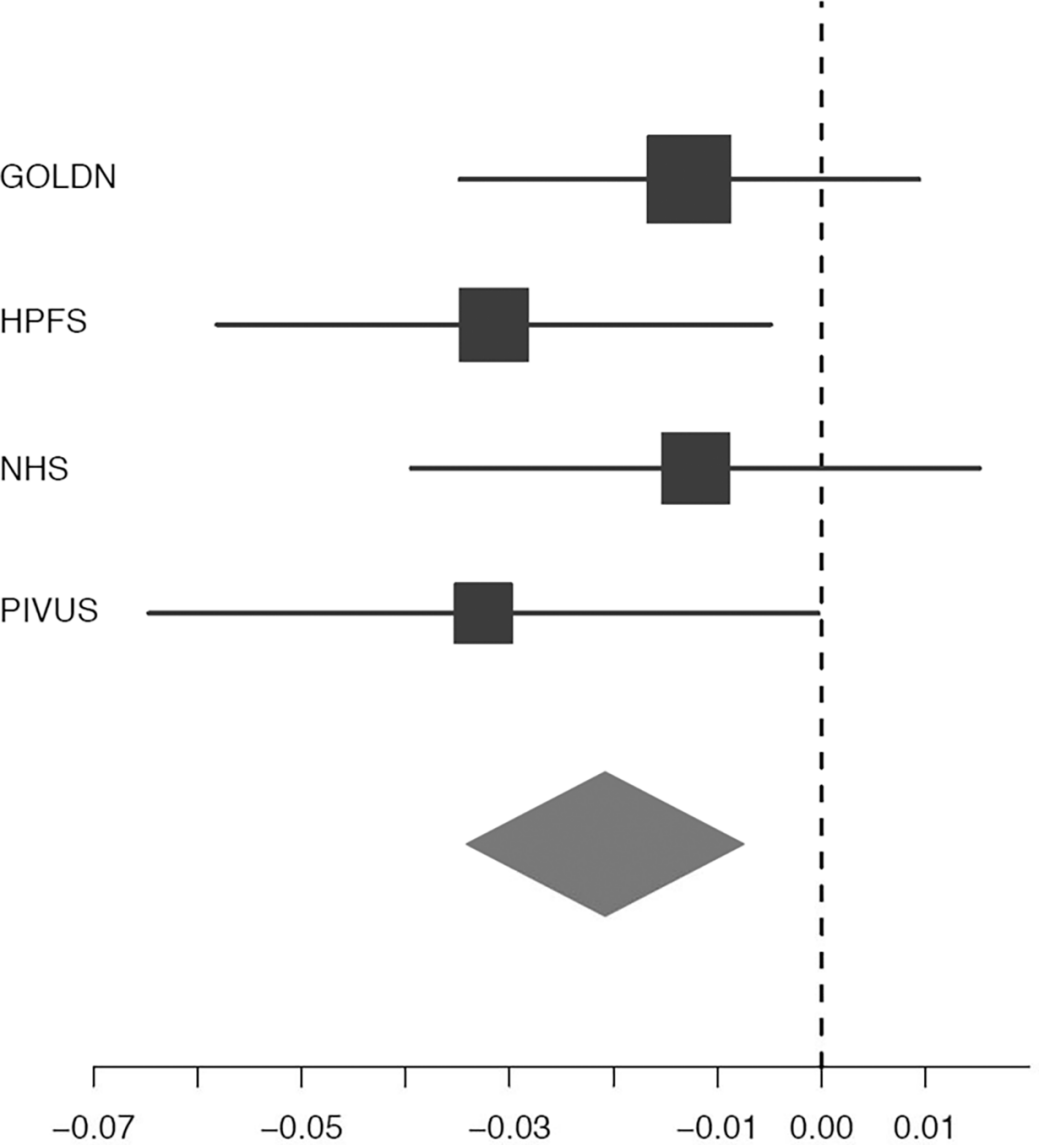
Change in percent units (95% CI) in circulating C17:0 levels for each copy of the C allele of rs11884924 (*LCT*). Although five cohorts had measures of C17:0 (CHS, GOLDN, HPFS, NHS, PIVUS), the CHS cohort did not have results for the SNP rs11884924 (LCT), and therefore is not included. Findings for rs16832067 (*LCT*) and rs3816088 (*LCT*) were similar in magnitude. The horizontal lines denote the 95% CIs and the squares represent the point estimate of each study. The size of the square is proportional to its inverse-variance weight in the meta-analysis. The diamond represents the pooled meta-analysis effect size estimate.

In order to evaluate the potential influence of genetic variation related to lactase activity on dairy consumption, we evaluated relations of 40 SNPs in *LCT* with levels of circulating C15:0 and C17:0, which are considered to be biomarkers of dairy or dairy fat consumption [3, 8, 9]. No significant associations were seen between any *LCT* SNP and C15:0 level (Table F in S1 Tables). In contrast, 3 SNPs in *LCT* were significantly associated with C17:0 level: rs11884924, rs16832067, and rs3816088 (FDR-adjusted P = 4.0×10^−2^; Table 2, Table G in S1 Tables). Each copy of the variant alleles in these SNPs was associated with approximately 0.02 unit lower C17:0 levels, with consistent direction of association for all four cohorts included in the analyses (**Fig 2**). No associations were observed for the combined sum of C15:0+C17:0 (Table H in S1 Tables).

### Tricosanoic acid (C23:0), *SPTLC3* and *CERS4* genes

Little is known about the metabolism of C23:0, a very long chain OCSFA. We examined correlations of C23:0 level with levels of other FA in the CHS cohort. We observed significant correlations of C23:0 level with the OCSFA C15:0 (r = 0.21, P = 6x10^-22^) and C17:0 (r = 0.33, P = 3x10^-55^). In addition, C23:0 level was strongly associated with VLSFA with even carbon numbers (r = 0.58 with C20:0 level, P = 1x10^-192^; 0.65 with C22:0 level, P = 4x10^-269^; and 0.57 with C24:0 level, P = 2x10^-19^). We recently reported genome wide associations of VLSFA with SNPs in two genes in the *de novo* synthesis pathway of ceramides and other sphingolipids, *SPTLC3* and *CERS4* [18]. Both VLSFA and C23:0 are components of sphingolipids [28, 29]. Thus, we investigated whether genetic variation in *SPTLC3* and *CERS4* was also associated with variation in C23:0 level. The SNPs associated with VLSFA were not associated with C23:0 levels (Table I in S1 Tables); however, 9 SNPs in *CERS4* were significantly associated with C23:0 level (FDR-adjusted P <5×10^−2^) (Table 2). There was no significant association between C23:0 level with SNPs in the *SPTLC3* gene (Table J in S1 Tables).

## Discussion

In this meta-analysis of 8 cohorts of adults of European ancestry, we found no evidence that common genetic variations are associated with circulating levels of C15:0 or C23:0 at genome wide significance threshold. Findings from C23:0 were limited to four cohorts with available data. We found one SNP in the *MYO10* gene associated with variation in circulating levels of C17:0, and in analysis limited to two cohorts with available data, two SNPs in the *DLEU* gene were associated with variation in levels of C19:0. The limited number of significant genome-wide associations suggests that circulating levels of OCSFA may be predominantly influenced by non-genetic factors. In contrast, using a candidate-gene approach, we found novel evidence that circulating C17:0 levels are associated with genetic variation in *LCT*, the gene responsible for adult-onset lactose intolerance [16]. In addition, C23:0 levels were associated with genetic variation in *CERS4*, a candidate gene involved in ceramide synthesis.

Located on chromosome 2, *LCT* is the single gene encoding the lactase enzyme, which regulates the hydrolysis of several molecules including the disaccharide lactose, the main carbohydrate in milk [16]. Characterized by reduced expression of the lactase enzyme in the intestine, lactase non-persistence leads to inability to digest milk lactose in over 50% adults worldwide [17]. The observed association of three HapMap SNPs in the *LCT* gene with C17:0 levels suggests that other genetic variants in the *LCT* gene could potentially influence consumption of dairy, which is one of the primary dietary sources of C17:0. With relatively modest correlations with dairy fat or dairy foods (r ranging between 0.16 and 0.40) [3, 8, 14], plasma levels of C15:0 and C17:0 have been used as objective biomarkers of dairy fat intake [3, 7, 8, 14, 15], although these FA also exist in seafood. The lack of significant associations between SNPs in the *LCT* gene with C15:0 may be partially attributed to potential differences in biologic processes related to FA absorption and incorporation into lipid fractions. For example, although the content of C17:0 in dairy fat is lower than that of C15:0, the C17:0 levels in plasma is about two times higher than that of C15:0 [30, 31]. It is also possible that differences in background diet, especially as it relates to other food sources contributing to circulating levels of C15:0 and C17:0 may vary, leading to differences in physiological response to changes in dairy consumption. Further work in needed to investigate how genetic variation in *LCT* could affect circulating levels of dairy-derived OCSFA, particularly C17:0.

Could circulating levels of OCSFA be influenced by endogenous metabolic processes? Although odd-chain FA are synthesized by the rumen bacterial flora and are known to derive predominantly from ruminant foods, recent studies in rodents reported that plasma phospholipid C15:0 and C17:0 may be endogenously produced by elongation of shorter OCSFA such as propionic acid (3:0) and heptanoic acid (7:0) [31, 32], or by α-oxidation of stearic acid (18:0) [33]. Whether such pathways contribute to C15:0 and C17:0 in humans is unknown. In prior investigations from this CHARGE consortium, we found multiple genetic variants associated with levels of FA known to be influenced by endogenous metabolism [18, 34–36], supporting the hypothesis of endogenous synthesis in humans. In contrast, we found no or little significant genome-wide associations with FA that cannot be synthesized endogenously by humans, e.g. *trans* fatty acids [37]. This suggests that circulating levels of OCSFA, are not appreciably influenced by genetic control, supporting primary influence of dietary sources of these FA.

Little is known of the metabolism and sources of circulating C23:0, in spite of the association of higher circulating levels with lower risk of diabetes in EPIC [38]. Sources of C23:0 may be both exogenous and endogenous. For example, C23:0 is found in milk, in gangliosides [39], although intestinal absorption of this particularly hydrophobic FA may be limited. In addition, the OCSFA C17:0 has been shown to be elongated to C23:0 in rat brain [40], suggesting the possibility of endogenous production of circulating C23:0. Possibly for this reason, we observed a modest correlation between C23:0 and C17:0. As true for other VLSFA, C23:0 is predominantly a component of sphingolipids, such as ceramides and sphingomyelins [28, 29]. Possibly for this reason, we saw an association of circulating C23:0 with common gene variation in *CERS4*, a ceramide synthase gene also associated with C20:0, 22:0 and C24:0 [18, 41, 42]. The two SNPs in *CERS4* reportedly associated with C20:0 in one direction, and with C22:0 and C24:0 in another direction [18], were not associated with C23:0. Instead, 9 other common SNPs were associated with C23:0 levels. Altogether, these findings raise the intriguing possibility that gene variation in *CERS4* may influence FA specificity of the enzyme, and that the resulting ceramide is destined at least in part for circulating sphingomyelin. In fact, it has been suggested that ceramides with VLSFA are prioritized for sphingomyelin production [43].

Our study has several strengths. The evaluation of genetic predictors of phospholipid OCSFA, reflecting both membrane and tissue phospholipids, across 8 cohorts provided the largest investigation of OCSFA to date among participants of European descent. We used both an agnostic approach and a hypothesis-based approach. We evaluated associations of C17:0 and C15:0 with SNPs in the *LCT*, and C23:0 with SNPs in *CERS4*, and *SPTLC3* genes, providing new insights on potential influence of adult-onset lactose intolerance and sphingolipid synthesis on circulating levels of C17:0 and C23:0 respectively.

Potential limitations should also be considered. Not all the studies had measured all the FA, limiting the sample size for some of the analyses. OCSFA are in small amounts in phospholipids and erythrocytes, representing less than 1% of total FA and we cannot discard the potential for type II error due to random measurement error associated with FA quantifying methods. This investigation focused on genetic associations, and the potential biological effects of the identified SNPs on circulating levels of OCSFA remain unknown. The SNPs associated with OCSFA are in high linkage disequilibrium with other SNPs in the region, and sequencing of the region is needed to identify potential causal variants. Finally, this analysis only included participants of European ancestry; further studies are needed to expand these findings to other ethnicities.

In conclusion, in this first GWAS investigation of OCSFA, we found no strong evidence for genetic control of circulating levels of these FA. Using a candidate-gene approach, we identified novel associations of genetic variants in the *LCT* gene associated with circulating level of C17:0. We also found that circulating level of C23:0 was associated with genetic variation in *CERS4*.

## Supporting information

S1 Tables Supplemental Tables A-J.
(DOCX)

S1 Text Supplemental text. (DOCX)
