# Supplementary material for: Genome-wide association meta-analysis of circulating odd-numbered chain saturated fatty acids: Results from the CHARGE Consortium
Source: PLoS One. 2018 May 8;13(5):e0196951. doi: 10.1371/journal.pone.0196951 (PMC5940220; doi:10.1371/journal.pone.0196951)
Supplement: S1 Tables — Supplemental Tables A-J. (DOCX) [file pone.0196951.s001.docx]

Table A. Top GWAS associations with circulating 15:0 in European-ancestry participants^1^

| Chromosome | SNP | Coded allele (frequency) | P value | Beta coefficient (SE) |
| --- | --- | --- | --- | --- |
| 18 | rs973730 | T/C (0.81) | 6.50×10^-7^ | -0.0039 (0.0008) |
| 18 | rs10502435 | T/C (0.19) | 6.55×10^-7^ | 0.0039 (0.0008) |
| 18 | rs12373434 | T/C (0.81) | 6.84×10^-7^ | -0.0039 (0.0008) |
| 18 | rs11662308 | T/G (0.81) | 7.10×10^-7^ | -0.0039 (0.0008) |
| 18 | rs11083274 | T/C (0.81) | 1.26×10^-6^ | -0.0039 (0.0008) |
| 4 | rs1824965 | T/C (0.46) | 1.33×10^-6^ | -0.0031 (0.0006) |
| 11 | rs17121057 | A/C (0.85) | 1.57×10^-6^ | -0.0291 (0.0061) |
| 18 | rs8093350 | A/G (0.20) | 1.94×10^-6^ | -0.0037 (0.0008) |
| 18 | rs2337110 | T/C (0.16) | 1.99×10^-6^ | -0.004 (0.0008) |
| 18 | rs11662721 | T/C (0.19) | 2.12×10^-6^ | 0.0038 (0.0008) |
| 14 | rs7140891 | T/C (0.86) | 2.24×10^-6^ | 0.0266 (0.0056) |
| 12 | rs11116498 | T/C (0.86) | 2.87×10^-6^ | 0.0045 (0.001) |
| 12 | rs10779113 | A/G (0.86) | 3.08×10^-6^ | 0.0045 (0.001) |
| 19 | rs4932739 | T/G (0.22) | 3.20×10^-6^ | -0.0347 (0.0075) |
| 18 | rs8086339 | T/G (0.19) | 3.60×10^-6^ | 0.0034 (0.0008) |

^1^ n=11,494 participants. Associations evaluated at genome-wide significance (alpha=5.0×10^-8^).

Table B Top GWAS associations with circulating 17:0 in European-ancestry participants^1^

| Chromosome | SNP | Coded allele (frequency) | P value | Beta coefficient (SE) |
| --- | --- | --- | --- | --- |
| 5 | rs13361131 | A/G (0.99) | 1.37×10^-8^ | -0.1346 (0.024) |
| 9 | rs17650729 | C/G (0.96) | 6.36×10^-8^ | -0.0324 (0.006) |
| 5 | rs17651415 | A/G (0.85) | 8.63×10^-7^ | -0.0112 (0.002) |
| 5 | rs7719940 | T/C (0.73) | 9.65 ×10^-7^ | -0.008 (0.002) |
| 13 | rs9315681 | A/C (0.76) | 1.04×10^-6^ | 0.0079 (0.002) |
| 13 | rs9566425 | A/G (0.76) | 1.07×10^-6^ | 0.0079 (0.002) |
| 13 | rs9576793 | C/G (0.25) | 1.09×10^-6^ | -0.0079 (0.002) |
| 13 | rs9566421 | A/G (0.75) | 1.15×10^-6^ | 0.0078 (0.002) |
| 13 | rs2880301 | T/C (0.25) | 1.20×10^-6^ | 0.0145 (0.003) |
| 13 | rs9576788 | A/G (0.25) | 1.22×10^-6^ | -0.0077 (0.002) |
| 7 | rs11561859 | T/C (0.90) | 1.55×10^-6^ | -0.0016 (0.002) |
| 17 | rs9905172 | T/C (0.81) | 1.75×10^-6^ | 0.017 (0.0035) |
| 5 | rs7736840 | T/C (0.72) | 2.26×10^-6^ | -0.0075 (0.002) |
| 4 | rs7690143 | T/G (0.99) | 2.54×10^-6^ | -0.0615 (0.013) |
| 5 | rs11133865 | T/C (0.73) | 3.60×10^-6^ | -0.0074 (0.0016) |

^1^ n=6,016 participants. Associations evaluated at genome-wide significance (alpha=5.0×10^-8^).

Table C. Top GWAS associations with circulating 15:0+17:0 European-ancestry participants^1^

| Chromosome | SNP | Coded allele (frequency) | P value | Beta coefficient (SE) |
| --- | --- | --- | --- | --- |
| 5 | rs7719940 | T/C (0.72) | 7.12×10^-8^ | -0.0148 (0.0027) |
| 11 | rs1490088 | T/C (0.97) | 1.04×10^-7^ | 0.0401 (0.0075) |
| 11 | rs11038755 | A/G (0.97) | 1.48×10^-7^ | 0.0422 (0.008) |
| 11 | rs11038756 | T/C (0.97) | 1.67×10^-7^ | 0.0423 (0.0081) |
| 5 | rs7736840 | T/C (0.72) | 1.82×10^-7^ | -0.0139 (0.0027) |
| 11 | rs11038747 | T/C (0.96) | 2.02×10^-7^ | 0.0427 (0.0082) |
| 5 | rs2625206 | T/C (0.69) | 2.58×10^-7^ | -0.0128 (0.0025) |
| 5 | rs11133865 | T/C (0.72) | 2.84×10^-7^ | -0.014 (0.0027) |
| 5 | rs17651415 | A/G (0.85) | 3.37×10^-7^ | -0.0197 (0.0039) |
| 11 | rs12363382 | C/G (0.97) | 3.85×10^-7^ | 0.0391 (0.0077) |
| 11 | rs11038764 | A/G (0.02) | 7.30×10^-7^ | -0.0544 (0.011) |
| 5 | rs13361131 | A/G (0.99) | 7.44×10^-7^ | -0.1598 (0.0323) |
| 11 | rs11038750 | A/G (0.96) | 3.29×10^-6^ | 0.0341 (0.0073) |
| 5 | rs10475324 | A/G (0.01) | 3.33×10^-6^ | 0.1029 (0.0221) |
| 20 | rs1182466 | T/C (0.66) | 4.52×10^-6^ | 0.0122 (0.0027) |

^1^ n=6,016 participants. Associations evaluated at genome-wide significance (alpha=5.0×10^-8^).

Table D. Top GWAS associations with circulating 19:0 in European-ancestry participants^1^

| Chromosome | SNP | Coded allele (frequency) | P value | Beta coefficient (SE) |
| --- | --- | --- | --- | --- |
| 13 | rs12874278 | T/C (0.06) | 7.07×10^-9^ | 0.0199 (0.0034) |
| 13 | rs17363566 | A/G (0.06) | 7.07×10^-9^ | 0.0199 (0.0034) |
| 13 | rs17074093 | A/G (0.95) | 1.31×10^-8^ | -0.0214 (0.0038) |
| 13 | rs12871645 | T/G (0.05) | 1.32×10^-8^ | 0.0213 (0.0037) |
| 13 | rs17074143 | A/T (0.95) | 1.32×10^-8^ | -0.0213 (0.0037) |
| 13 | rs17074145 | C/G (0.05) | 1.32×10^-8^ | 0.0213 (0.0037) |
| 13 | rs11842790 | T/C (0.05) | 1.36 ×10^-8^ | 0.0213 (0.0037) |
| 13 | rs12853498 | A/T (0.05) | 1.39×10^-8^ | 0.0213 (0.0037) |
| 13 | rs12874827 | T/G (0.95) | 1.49×10^-8^ | -0.0212 (0.0038) |
| 13 | rs706603 | T/C (0.95) | 2.47×10^-8^ | -0.0201 (0.0036) |
| 13 | rs4770891 | C/G (0.96) | 1.15×10^-7^ | -0.0276 (0.0052) |
| 7 | rs13221923 | A/G (0.02) | 1.83×10^-7^ | 0.0359 (0.0069) |
| 7 | rs1468510 | A/G (0.98) | 1.89×10^-7^ | -0.0358 (0.0069) |
| 7 | rs13226693 | T/C (0.01) | 1.91×10^-7^ | 0.0363 (0.007) |
| 7 | rs12703118 | A/G (0.99) | 4.19×10^-7^ | -0.0393 (0.0078) |

^1^ n=1,910 participants. Associations evaluated at genome-wide significance (alpha=5.0×10^-8^).

Table E. Top GWAS associations with circulating 23:0 in European-ancestry participants^1^

| Chromosome | SNP | Coded allele (frequency) | P value | Beta coefficient (SE) |
| --- | --- | --- | --- | --- |
| 1 | rs16850871 | A/G (0.96) | 1.11×10^-7^ | -0.0164 (0.0031) |
| 4 | rs17003427 | T/C (0.05) | 1.44×10^-7^ | -0.0147 (0.0028) |
| 1 | rs7513659 | A/G (0.04) | 1.50×10^-7^ | 0.0164 (0.0031) |
| 4 | rs9992277 | A/G (0.05) | 2.96×10^-7^ | -0.0141 (0.0028) |
| 4 | rs12648219 | A/G (0.95) | 3.02×10^-7^ | 0.0143 (0.0028) |
| 4 | rs6839280 | T/C (0.95) | 3.04×10^-7^ | 0.0143 (0.0028) |
| 4 | rs6857679 | T/C (0.05) | 3.05×10^-7^ | -0.0143 (0.0028) |
| 4 | rs7439893 | A/G (0.95) | 3.06×10^-7^ | 0.0143 (0.0028) |
| 4 | rs727761 | A/C (0.95) | 3.08×10^-7^ | 0.0142 (0.0028) |
| 4 | rs10518201 | A/G (0.95) | 3.53×10^-7^ | 0.0141 (0.0028) |
| 4 | rs1978552 | T/G (0.05) | 4.11×10^-7^ | -0.0141 (0.0028) |
| 4 | rs727760 | A/G (0.95) | 4.17×10^-7^ | 0.0141 (0.0028) |
| 4 | rs17003443 | T/C (0.05) | 4.89×10^-7^ | -0.0141 (0.0028) |
| 4 | rs4432766 | A/T (0.05) | 6.17×10^-7^ | -0.0141 (0.0028) |
| 4 | rs10518202 | T/G (0.05) | 6.99×10^-7^ | -0.0141 (0.0028) |

^1^n=7,582 participants. Associations evaluated at genome-wide significance (alpha=5.0×10^-8^).

Table F. Top 40 associations of common SNPs in the lactase (LCT) gene^1^ with circulating 15:0 in European-ancestry participants^2^

| SNP | Coded allele (frequency) | Unadjusted  p-value | FDR-adjusted  p-value | Beta coefficient (SE) |
| --- | --- | --- | --- | --- |
| rs1807356 | T/C (0.86) | 0.051 | 3.5 ×10^-1^ | 0.0019 (0.001) |
| rs4954633 | T/C (0.09) | 0.063 | 3.5 ×10^-1^ | -0.0022 (0.001) |
| rs4954430 | T/C (0.09) | 0.094 | 3.5 ×10^-1^ | -0.002 (0.001) |
| rs4954445 | A/T (0.11) | 0.11 | 3.5 ×10^-1^ | -0.0017 (0.001) |
| rs892715 | T/C (0.29) | 0.12 | 3.5 ×10^-1^ | -0.0012 (0.001) |
| rs2164210 | T/C (0.30) | 0.12 | 3.5 ×10^-1^ | -0.0012 (0.001) |
| rs6719488 | T/G (0.71) | 0.12 | 3.5 ×10^-1^ | 0.0012 (0.001) |
| rs3769011 | A/T (0.89) | 0.12 | 3.5 ×10^-1^ | 0.0017 (0.001) |
| rs3739022 | A/G (0.11) | 0.12 | 3.5 ×10^-1^ | -0.0017 (0.001) |
| rs872151 | T/C (0.11) | 0.12 | 3.5 ×10^-1^ | -0.0016 (0.001) |
| rs12992815 | C/G (0.89) | 0.12 | 3.5 ×10^-1^ | 0.0017 (0.001) |
| rs2874874 | A/C (0.89) | 0.13 | 3.5 ×10^-1^ | 0.0016 (0.001) |
| rs748841 | T/C (0.71) | 0.13 | 3.5 ×10^-1^ | 0.0012 (0.001) |
| rs7579771 | A/T (0.29) | 0.13 | 3.5 ×10^-1^ | -0.0012 (0.001) |
| rs1011361 | A/G (0.73) | 0.13 | 3.5 ×10^-1^ | 0.0012 (0.001) |
| rs745500 | A/G (0.71) | 0.14 | 3.5 ×10^-1^ | 0.0011 (0.001) |
| rs2322660 | T/C (0.71) | 0.15 | 3.5 ×10^-1^ | 0.0011 (0.001) |
| rs3754690 | T/C (0.11) | 0.16 | 3.6 ×10^-1^ | -0.0015 (0.001) |
| rs11884924 | A/C (0.04) | 0.22 | 4.4 ×10^-1^ | -0.0029 (0.002) |
| rs2322659 | T/C (0.27) | 0.22 | 4.4 ×10^-1^ | -0.0009 (0.001) |
| rs16832067 | A/G (0.96) | 0.23 | 4.4 ×10^-1^ | 0.0028 (0.002) |
| rs3816088 | C/G (0.04) | 0.25 | 4.4 ×10^-1^ | -0.0027 (0.002) |
| rs2278544 | A/G (0.25) | 0.29 | 5.0 ×10^-1^ | -0.0008 (0.001) |
| rs2236783 | A/G (0.75) | 0.33 | 5.1 ×10^-1^ | 0.0008 (0.001) |
| rs3754691 | T/C (0.91) | 0.51 | 8.1 ×10^-1^ | -0.0031 (0.005) |
| rs2304370 | A/G (0.19) | 0.65 | 9.6 ×10^-1^ | -0.0004 (0.001) |
| rs2304371 | A/G (0.81) | 0.65 | 9.6 ×10^-1^ | 0.0004 (0.001) |
| rs12475516 | C/G (0.81) | 0.67 | 9.6 ×10^-1^ | 0.0004 (0.001) |
| rs2322813 | A/G (0.84) | 0.80 | 9.9 ×10^-1^ | -0.0003 (0.001) |
| rs2015532 | T/G (0.83) | 0.82 | 9.9 ×10^-1^ | 0.0002 (0.001) |
| rs3213890 | A/G (0.17) | 0.89 | 9.9 ×10^-1^ | 0.0001 (0.001) |
| rs746857 | A/G (0.83) | 0.90 | 9.9 ×10^-1^ | 0.0001 (0.001) |
| rs3754689 | T/C (0.17) | 0.92 | 9.9 ×10^-1^ | -0.0001 (0.001) |
| rs1042712 | C/G (0.17) | 0.96 | 9.9 ×10^-1^ | 0 (0.001) |
| rs9636213 | A/G (0.83) | 0.97 | 9.9 ×10^-1^ | 0 (0.001) |
| rs730005 | T/C (0.83) | 0.97 | 9.9 ×10^-1^ | 0 (0.001) |
| rs3769013 | T/C (0.17) | 0.98 | 9.9 ×10^-1^ | 0 (0.001) |
| rs12373779 | A/C (0.83) | 0.99 | 9.9 ×10^-1^ | 0 (0.001) |
| rs3769008 | T/C (0.18) | 0.99 | 9.9 ×10^-1^ | 0 (0.001) |
| rs3769012 | A/G (0.17) | 0.995 | 9.9 ×10^-1^ | 0 (0.001) |

^1^ located in Chromosome 2

^2^ n=11,494 participants. Associations evaluated at genome-wide significance (alpha=5.0×10^-8^).

Table G. Top 40 associations of common SNPs in the lactase (*LCT*) gene^1^ with circulating 17:0 in European-ancestry participants^2^

| SNP | Coded allele (frequency) | Unadjusted  p-value | FDR-adjusted  p-value | Beta coefficient (SE) |
| --- | --- | --- | --- | --- |
| rs11884924 | A/C (0.02) | 0.002 | 4.3 ×10^-2^ | -0.0208 (0.007) |
| rs16832067 | A/G (0.98) | 0.003 | 4.3 ×10^-2^ | 0.0204 (0.007) |
| rs3816088 | C/G (0.02) | 0.003 | 4.3 ×10^-2^ | -0.0201 (0.007) |
| rs1807356 | T/C (0.86) | 0.09 | 7.5 ×10^-1^ | 0.0035 (0.002) |
| rs892715 | T/C (0.27) | 0.23 | 7.5 ×10^-1^ | -0.0021 (0.002) |
| rs6719488 | T/G (0.73) | 0.24 | 7.5 ×10^-1^ | 0.0021 (0.002) |
| rs872151 | T/C (0.11) | 0.24 | 7.5 ×10^-1^ | -0.0025 (0.002) |
| rs7579771 | A/T (0.27) | 0.24 | 7.5 ×10^-1^ | -0.002 (0.002) |
| rs2164210 | T/C (0.29) | 0.25 | 7.5 ×10^-1^ | -0.002 (0.002) |
| rs745500 | A/G (0.73) | 0.25 | 7.5 ×10^-1^ | 0.002 (0.002) |
| rs748841 | T/C (0.72) | 0.26 | 7.5 ×10^-1^ | 0.002 (0.002) |
| rs2322660 | T/C (0.72) | 0.29 | 7.5 ×10^-1^ | 0.0019 (0.002) |
| rs4954430 | T/C (0.08) | 0.35 | 7.5 ×10^-1^ | -0.0023 (0.002) |
| rs1011361 | A/G (0.74) | 0.35 | 7.5 ×10^-1^ | 0.0016 (0.002) |
| rs3754691 | T/C (0.99) | 0.35 | 7.5 ×10^-1^ | 0.009 (0.01) |
| rs4954445 | A/T (0.1) | 0.36 | 7.5 ×10^-1^ | -0.002 (0.002) |
| rs3769011 | A/T (0.9) | 0.38 | 7.5 ×10^-1^ | 0.0019 (0.002) |
| rs12992815 | C/G (0.9) | 0.39 | 7.5 ×10^-1^ | 0.0019 (0.002) |
| rs3739022 | A/G (0.1) | 0.39 | 7.5 ×10^-1^ | -0.0019 (0.002) |
| rs2874874 | A/C (0.89) | 0.40 | 7.5 ×10^-1^ | 0.0018 (0.002) |
| rs4954633 | T/C (0.08) | 0.41 | 7.5 ×10^-1^ | -0.0019 (0.002) |
| rs3754690 | T/C (0.11) | 0.41 | 7.5 ×10^-1^ | -0.0018 (0.002) |
| rs2304371 | A/G (0.82) | 0.50 | 8.1 ×10^-1^ | 0.0017 (0.003) |
| rs2304370 | A/G (0.18) | 0.51 | 8.1 ×10^-1^ | -0.0017 (0.003) |
| rs12475516 | C/G (0.82) | 0.51 | 8.1 ×10^-1^ | 0.0017 (0.003) |
| rs2236783 | A/G (0.76) | 0.53 | 8.1 ×10^-1^ | 0.0012 (0.002) |
| rs2322659 | T/C (0.25) | 0.60 | 8.7 ×10^-1^ | -0.0009 (0.002) |
| rs2278544 | A/G (0.24) | 0.66 | 8.7 ×10^-1^ | -0.0008 (0.002) |
| rs2322813 | A/G (0.85) | 0.70 | 8.7 ×10^-1^ | -0.001 (0.003) |
| rs3769013 | T/C (0.16) | 0.72 | 8.7 ×10^-1^ | 0.0009 (0.003) |
| rs746857 | A/G (0.85) | 0.81 | 8.7 ×10^-1^ | -0.0005 (0.002) |
| rs730005 | T/C (0.84) | 0.82 | 8.7 ×10^-1^ | -0.0006 (0.003) |
| rs3213890 | A/G (0.15) | 0.82 | 8.7 ×10^-1^ | 0.0005 (0.002) |
| rs1042712 | C/G (0.16) | 0.83 | 8.7 ×10^-1^ | 0.0005 (0.002) |
| rs12373779 | A/C (0.84) | 0.83 | 8.7 ×10^-1^ | -0.0005 (0.003) |
| rs3769008 | T/C (0.16) | 0.83 | 8.7 ×10^-1^ | 0.0005 (0.003) |
| rs9636213 | A/G (0.84) | 0.83 | 8.7 ×10^-1^ | -0.0005 (0.003) |
| rs3769012 | A/G (0.15) | 0.85 | 8.7 ×10^-1^ | 0.0005 (0.003) |
| rs2015532 | T/G (0.85) | 0.86 | 8.7 ×10^-1^ | 0.0004 (0.002) |
| rs3754689 | A/G (0.16) | 0.87 | 8.7 ×10^-1^ | 0.0004 (0.002) |

^1^located in Chromosome 2

^2^ n=6,016 participants. Associations evaluated at genome-wide significance (alpha=5.0×10^-8^).

Table H. Top 40 associations of common SNPs in the lactase (*LCT*) gene^1^ with circulating 15:0+17:0 in European-ancestry participants

| SNP | Coded allele (frequency) | Unadjusted  p-value | FDR-adjusted  p-value | Beta coefficient (SE) |
| --- | --- | --- | --- | --- |
| rs16832067 | A/G (0.98) | 0.005 | 1.2 ×10^-1^ | 0.0284 (0.01) |
| rs3816088 | C/G (0.02) | 0.008 | 1.2 ×10^-1^ | -0.0268 (0.01) |
| rs11884924 | A/C (0.02) | 0.009 | 1.2 ×10^-1^ | -0.0267 (0.01) |
| rs1011361 | A/G (0.73) | 0.16 | 5.9 ×10^-1^ | 0.0042 (0.003) |
| rs892715 | T/C (0.28) | 0.16 | 5.9 ×10^-1^ | -0.0043 (0.003) |
| rs6719488 | T/G (0.72) | 0.17 | 5.9 ×10^-1^ | 0.0042 (0.003) |
| rs7579771 | A/T (0.28) | 0.17 | 5.9 ×10^-1^ | -0.0041 (0.003) |
| rs748841 | T/C (0.72) | 0.18 | 5.9 ×10^-1^ | 0.0041 (0.003) |
| rs745500 | A/G (0.72) | 0.18 | 5.9 ×10^-1^ | 0.0041 (0.003) |
| rs2164210 | T/C (0.29) | 0.18 | 5.9 ×10^-1^ | -0.0041 (0.003) |
| rs2304371 | A/G (0.81) | 0.19 | 5.9 ×10^-1^ | 0.0061 (0.005) |
| rs12475516 | C/G (0.81) | 0.19 | 5.9 ×10^-1^ | 0.006 (0.005) |
| rs2304370 | A/G (0.19) | 0.20 | 5.9 ×10^-1^ | -0.0059 (0.005) |
| rs2322660 | T/C (0.72) | 0.21 | 5.9 ×10^-1^ | 0.0039 (0.003) |
| rs2015532 | T/G (0.85) | 0.23 | 6.0 ×10^-1^ | 0.0048 (0.004) |
| rs2278544 | A/G (0.25) | 0.26 | 6.4 ×10^-1^ | -0.0033 (0.003) |
| rs1807356 | T/C (0.85) | 0.28 | 6.7 ×10^-1^ | 0.0037 (0.004) |
| rs4954430 | T/C (0.08) | 0.33 | 6.8 ×10^-1^ | -0.0039 (0.004) |
| rs2322659 | T/C (0.25) | 0.37 | 6.8 ×10^-1^ | -0.0027 (0.003) |
| rs2236783 | A/G (0.77) | 0.40 | 6.8 ×10^-1^ | 0.0028 (0.003) |
| rs4954633 | T/C (0.08) | 0.42 | 6.8 ×10^-1^ | -0.0031 (0.004) |
| rs746857 | A/G (0.85) | 0.44 | 6.8 ×10^-1^ | 0.003 (0.004) |
| rs872151 | T/C (0.11) | 0.46 | 6.8 ×10^-1^ | -0.0027 (0.004) |
| rs1042712 | C/G (0.16) | 0.49 | 6.8 ×10^-1^ | -0.0024 (0.004) |
| rs3769012 | A/G (0.16) | 0.50 | 6.8 ×10^-1^ | -0.0033 (0.005) |
| rs3213890 | A/G (0.16) | 0.52 | 6.8 ×10^-1^ | -0.0025 (0.004) |
| rs4954445 | A/T (0.1) | 0.54 | 6.8 ×10^-1^ | -0.0022 (0.004) |
| rs3754689 | A/G (0.16) | 0.55 | 6.8 ×10^-1^ | -0.0026 (0.004) |
| rs3769011 | A/T (0.9) | 0.56 | 6.8 ×10^-1^ | 0.0021 (0.004) |
| rs3739022 | A/G (0.1) | 0.57 | 6.8 ×10^-1^ | -0.0021 (0.004) |
| rs12992815 | C/G (0.9) | 0.57 | 6.8 ×10^-1^ | 0.0021 (0.004) |
| rs2874874 | A/C (0.89) | 0.58 | 6.8 ×10^-1^ | 0.002 (0.004) |
| rs3754691 | T/C (0.99) | 0.58 | 6.8 ×10^-1^ | -0.0075 (0.014) |
| rs3754690 | T/C (0.1) | 0.60 | 6.8 ×10^-1^ | -0.0019 (0.004) |
| rs9636213 | A/G (0.83) | 0.61 | 6.8 ×10^-1^ | 0.0024 (0.005) |
| rs12373779 | A/C (0.83) | 0.63 | 6.8 ×10^-1^ | 0.0023 (0.005) |
| rs3769008 | T/C (0.17) | 0.64 | 6.8 ×10^-1^ | -0.0023 (0.005) |
| rs730005 | T/C (0.83) | 0.66 | 6.8 ×10^-1^ | 0.0021 (0.005) |
| rs3769013 | T/C (0.17) | 0.66 | 6.8 ×10^-1^ | -0.0021 (0.005) |
| rs2322813 | A/G (0.84) | 0.698 | 7.0 ×10^-1^ | 0.002 (0.005) |

^1^ located in Chromosome 2

^2^ n=6,016 participants. Associations evaluated at genome-wide significance (alpha=5.0×10^-8^)

Table I. Top 40 associations of SNPs in *LASS4/CERS4^1^* gene with circulating 23:0 in European-ancestry participants^2^

| SNP | Coded allele (frequency) | Unadjusted  p-value | FDR-adjusted  p-value | Beta coefficient (SE) |
| --- | --- | --- | --- | --- |
| rs36251 | C/G (0.55) | 0.002 | 3.0 ×10^-2^ | 0.005 (0.002) |
| rs10409603 | A/G (0.44) | 0.002 | 3.0 ×10^-2^ | -0.0041 (0.001) |
| rs2927718 | C/G (0.42) | 0.003 | 3.0 ×10^-2^ | 0.0036 (0.001) |
| rs1115199 | T/C (0.48) | 0.003 | 3.0 ×10^-2^ | -0.0041 (0.001) |
| rs367443 | A/G (0.46) | 0.004 | 3.0 ×10^-2^ | -0.004 (0.001) |
| rs2306199 | T/G (0.58) | 0.004 | 3.0 ×10^-2^ | -0.0042 (0.002) |
| rs36249 | A/G (0.44) | 0.01 | 3.0 ×10^-2^ | 0.004 (0.001) |
| rs36258 | A/G (0.53) | 0.01 | 3.0 ×10^-2^ | 0.0045 (0.002) |
| rs17160348 | T/C (0.16) | 0.01 | 3.0 ×10^-2^ | -0.0069 (0.003) |
| rs36259 | A/G (0.77) | 0.02 | 8.0 ×10^-2^ | 0.0049 (0.002) |
| rs17160331 | C/G (0.27) | 0.02 | 9.0 ×10^-2^ | -0.0037 (0.002) |
| rs12891 | C/G (0.45) | 0.03 | 1.0 ×10^-1^ | 0.0036 (0.002) |
| rs1044547 | T/C (0.17) | 0.03 | 1.0 ×10^-1^ | -0.0054 (0.003) |
| rs10426096 | A/C (0.83) | 0.06 | 2.0 ×10^-1^ | 0.0035 (0.002) |
| rs17160332 | A/G (0.05) | 0.08 | 2.5 ×10^-1^ | 0.0051 (0.003) |
| rs11666971 | T/C (0.77) | 0.10 | 2.8 ×10^-1^ | 0.0028 (0.002) |
| rs36257 | A/T (0.72) | 0.16 | 4.2 ×10^-1^ | 0.0026 (0.002) |
| rs2967625 | A/G (0.51) | 0.17 | 4.2 ×10^-1^ | 0.0025 (0.002) |
| rs1124626 | A/G (0.26) | 0.25 | 5.8 ×10^-1^ | -0.0018 (0.002) |
| rs36247 | T/C (0.69) | 0.28 | 6.3 ×10^-1^ | 0.0017 (0.002) |
| rs11666913 | C/G (0.42) | 0.30 | 6.3 ×10^-1^ | 0.002 (0.002) |
| rs12973288 | T/C (0.04) | 0.31 | 6.3 ×10^-1^ | 0.0031 (0.003) |
| rs12986087 | A/C (0.96) | 0.32 | 6.3 ×10^-1^ | -0.0031 (0.003) |
| rs17160333 | T/C (0.84) | 0.36 | 6.7 ×10^-1^ | 0.0015 (0.002) |
| rs11666866 | A/G (0.43) | 0.37 | 6.7 ×10^-1^ | 0.0017 (0.002) |
| rs2913987 | A/C (0.18) | 0.40 | 6.7 ×10^-1^ | -0.0017 (0.002) |
| rs1466448 | A/C (0.8) | 0.41 | 6.7 ×10^-1^ | 0.0012 (0.002) |
| rs12974053 | T/C (0.09) | 0.44 | 6.7 ×10^-1^ | 0.0023 (0.003) |
| rs2927714 | T/C (0.2) | 0.44 | 6.7 ×10^-1^ | -0.0015 (0.002) |
| rs11672652 | T/C (0.14) | 0.45 | 6.7 ×10^-1^ | -0.0014 (0.002) |
| rs12610250 | A/G (0.47) | 0.48 | 7.0 ×10^-1^ | 0.0024 (0.003) |
| rs36248 | T/C (0.17) | 0.50 | 7.0 ×10^-1^ | -0.0016 (0.002) |
| rs6603157 | A/G (0.16) | 0.60 | 8.2 ×10^-1^ | -0.0008 (0.002) |
| rs2967622 | A/G (0.04) | 0.66 | 8.7 ×10^-1^ | 0.0015 (0.003) |
| rs17160311 | A/G (0.03) | 0.68 | 8.8 ×10^-1^ | 0.0013 (0.003) |
| rs2100944 | A/G (0.78) | 0.71 | 8.8 ×10^-1^ | 0.0007 (0.002) |
| rs379983 | A/G (0.04) | 0.73 | 8.9 ×10^-1^ | 0.0013 (0.004) |
| rs1466447 | T/C (0.83) | 0.77 | 9.2 ×10^-1^ | 0.0005 (0.002) |
| rs1542538 | T/C (0.14) | 0.80 | 9.2 ×10^-1^ | 0.0005 (0.002) |
| rs2967623 | A/T (0.14) | 0.826 | 9.3 ×10^-1^ | 0.0004 (0.002) |

^1^ located in Chromosome 19

^2^ n=7,582 participants. Associations evaluated at genome-wide significance (alpha=5.0×10^-8^).

Table J. Top 40 associations of SNPs in *SPTLC3*^1^ gene with circulating 23:0 in European-ancestry participants^2^

| SNP | Coded allele (frequency) | Unadjusted  p-value | FDR-adjusted  p-value | Beta coefficient (SE) |
| --- | --- | --- | --- | --- |
| rs6078937 | T/G (0.67) | 0.007 | 3.5 ×10^-1^ | 0.0034 (0.001) |
| rs4431013 | C/G (0.67) | 0.007 | 3.5 ×10^-1^ | 0.0034 (0.001) |
| rs11906407 | A/G (0.06) | 0.007 | 3.5 ×10^-1^ | -0.0068 (0.003) |
| rs11908149 | T/G (0.06) | 0.008 | 3.5 ×10^-1^ | -0.0068 (0.003) |
| rs6105044 | A/T (0.31) | 0.008 | 3.5 ×10^-1^ | -0.0034 (0.001) |
| rs6105042 | T/C (0.07) | 0.010 | 3.5 ×10^-1^ | -0.0063 (0.003) |
| rs6033625 | T/G (0.31) | 0.01 | 3.5 ×10^-1^ | -0.0033 (0.001) |
| rs2073303 | C/G (0.33) | 0.01 | 3.5 ×10^-1^ | -0.0032 (0.001) |
| rs6078938 | T/C (0.85) | 0.01 | 3.7 ×10^-1^ | -0.0041 (0.002) |
| rs7264061 | A/C (0.83) | 0.02 | 4.2 ×10^-1^ | -0.0038 (0.002) |
| rs6109699 | A/G (0.06) | 0.03 | 6.3 ×10^-1^ | -0.0058 (0.003) |
| rs6109708 | A/G (0.94) | 0.03 | 6.5 ×10^-1^ | 0.0057 (0.003) |
| rs16993768 | A/G (0.06) | 0.04 | 7.4 ×10^-1^ | -0.0054 (0.003) |
| rs882302 | A/C (0.06) | 0.05 | 8.0 ×10^-1^ | -0.0051 (0.003) |
| rs3910137 | T/C (0.94) | 0.05 | 8.0 ×10^-1^ | 0.0052 (0.003) |
| rs6074564 | A/T (0.88) | 0.05 | 8.0 ×10^-1^ | -0.0035 (0.002) |
| rs8119743 | A/G (0.12) | 0.07 | 8.0 ×10^-1^ | 0.0032 (0.002) |
| rs2236124 | T/C (0.88) | 0.07 | 8.0 ×10^-1^ | -0.0033 (0.002) |
| rs6109723 | A/G (0.88) | 0.07 | 8.0 ×10^-1^ | -0.0032 (0.002) |
| rs6078936 | A/C (0.88) | 0.07 | 8.0 ×10^-1^ | -0.0032 (0.002) |
| rs6078931 | T/C (0.17) | 0.07 | 8.0 ×10^-1^ | 0.0028 (0.002) |
| rs6109693 | T/C (0.05) | 0.07 | 8.0 ×10^-1^ | -0.005 (0.003) |
| rs6033633 | T/C (0.95) | 0.09 | 8.0 ×10^-1^ | -0.005 (0.003) |
| rs6033599 | A/G (0.85) | 0.10 | 8.0 ×10^-1^ | 0.0028 (0.002) |
| rs1321255 | T/G (0.95) | 0.10 | 8.0 ×10^-1^ | -0.0048 (0.003) |
| rs6033600 | A/G (0.15) | 0.10 | 8.0 ×10^-1^ | -0.0027 (0.002) |
| rs6033601 | A/G (0.85) | 0.10 | 8.0 ×10^-1^ | 0.0027 (0.002) |
| rs6041817 | A/G (0.85) | 0.11 | 8.0 ×10^-1^ | 0.0027 (0.002) |
| rs6109674 | A/C (0.15) | 0.11 | 8.0 ×10^-1^ | -0.0027 (0.002) |
| rs4814194 | T/G (0.16) | 0.11 | 8.0 ×10^-1^ | -0.0025 (0.002) |
| rs6041818 | A/G (0.15) | 0.11 | 8.0 ×10^-1^ | -0.0027 (0.002) |
| rs6041820 | T/G (0.15) | 0.11 | 8.0 ×10^-1^ | -0.0027 (0.002) |
| rs4814184 | A/C (0.13) | 0.11 | 8.0 ×10^-1^ | -0.0028 (0.002) |
| rs6041822 | C/G (0.15) | 0.11 | 8.0 ×10^-1^ | -0.0027 (0.002) |
| rs1431434 | T/G (0.85) | 0.11 | 8.0 ×10^-1^ | 0.0027 (0.002) |
| rs1431436 | T/G (0.85) | 0.11 | 8.0 ×10^-1^ | 0.0027 (0.002) |
| rs6041927 | A/G (0.95) | 0.12 | 8.0 ×10^-1^ | -0.0046 (0.003) |
| rs6041897 | T/G (0.54) | 0.12 | 8.0 ×10^-1^ | -0.0019 (0.001) |
| rs243881 | T/C (0.56) | 0.13 | 8.0 ×10^-1^ | 0.0018 (0.001) |
| rs243884 | A/G (0.44) | 0.13 | 8.0 ×10^-1^ | -0.0018 (0.001) |

^1^ located in Chromosome 20

^2^ n=7,582 participants. Associations evaluated at genome-wide significance (alpha=5.0×10^-8^).
